# Software review: Pony GE2

**DOI:** 10.1007/s10710-021-09409-5

**Published:** 2021-07-22

**Authors:** Tuong Manh Vu

**Affiliations:** University of Sheffield

## Introduction

PonyGE2 is an open source Python implementation of Grammatical Evolution, a Genetic Programming algorithm which uses formal grammars in the genotype to phenotype mapping to guide the search process [1]. It is developed and maintained by the Natural Computing Research and Applications group at University College Dublin. The preceding version PonyGE, released in 2010, was designed to be small and compact with a single source file. To accommodate the growing requirements for more functionality, a feature-rich and modular addition, PonyGE2, was released in 2017. PonyGE2 v0.2.0 is available from a GitHub repository (github.com/PonyGE/PonyGE2) and licensed for use under the GPL version 3.

## Strengths

PonyGE2 is freely available, lightweight, and easy to use. The download itself is 11 megabytes. PonyGE2 requires Python 3.5 and a few libraries: matplotlib, numpy, scipy, sklearn, pandas. There is no graphical user interface and instead PonyGE2 is run via the command line.

Documentation is vital to open source software. PonyGE2 has a conference paper [1] that gives a good overview of the software: the organizational structure of the code base, the control flow, grammar parsing, linear representation, tree representation, operators, etc. Additionally, PonyGE2 has detailed documentation which is hosted on the wiki pages of GitHub. The wiki pages explain in detail each aspect of the PonyGE2 software libraries and how to customize them to solve your own problem. For new users, there is a wide variety of examples: string-match, regression, classification, Pymax, integer sequence match, program synthesis, genetic improvement of regular expressions, and multi-objective optimisation. These examples can be executed just by changing the parameter file on the command-line. I encourage new users to use these examples to understand PonyGE2 as well as a starting point for their own problem.

PonyGE2 uses text-based files for grammar definition and parameter configuration. This allows the user to completely change the search algorithm with a few adjustments to the grammar or parameter files. For a new problem, developers need to specify a grammar file in BNF (Backus-Naur Form) format, a fitness function in Python, a new parameter configuration file, and a new dataset if it is a supervised learning problem. There are many existing fitness functions that can be re-used. Thus many problems will require little programming; just new grammar and parameter files.

PonyGE2 supports multi-core evaluation for Unix-like operating systems that speeds up the search process, especially when using high performance computing. Another useful feature is that PonyGE2 supports two types of representation for every individual: linear genome and derivation tree. Genetic operators for both representations can be mixed in a set-up since they are fully compatible with each other. Advantages and drawbacks of each type are discussed in [1]. For example, manipulating derivation tree reduces the number of invalid individuals and allows context-aware operations, but increases computational run-time.

## Weaknesses

The development team can be contacted by creating a new issue on the GitHub page. However, there is a need for a better tool for community participation. For example, a mailing list would be a better place for news, discussion, or peer support between PonyGE2 users.

The current version of PonyGE2 supports multi-objective optimisation using NSGA-II. NSGA-II is now two decades old and is only suitable for problems with two or three objectives. More modern algorithms [2] should be included from a wider choice of paradigms, e.g. decomposition-based algorithms such as MOEA/D, indicator-based algorithms such as SMS-EMOA, and multi-objective Bayesian optimizers such as ParEGO.

PonyGE2 only supports termination of the evolutionary process after a pre-specified number of generations. It should allow for other termination criteria, e.g. based on performance metrics such as hypervolume.

The current workflow set-up can be a barrier for many new users. The users need to download the source code and run it using the command line. Firstly, it would be nice if PonyGE2 was pip installable. Another suggestion is to create an interface to Jupyter Notebook, which is very popular with Python users. A quick-start guide would also be useful. Currently, users need to read the whole wiki because the section with examples and steps to add new problems is near the end of the documentation.

Lastly, the structure of PonyGE2 distributes the files of a new problem into multiple folders of the source code: grammars, fitness, datasets, parameters. If users only want to share their problem and optimizer configuration, they need to distribute just these files. To reproduce the work of others, users then need to deposit these files across the relevant folders. The file and project management aspects should be improved to ease replicability and reproducibility.

## Conclusion

I would recommend PonyGE2 to newcomers to Grammatical Evolution. The code is well-written and there is a variety of working examples. The developer team are very active on GitHub. Even though there is still room for further improvement and development, I think it is more important to focus on fostering a community of users to increase the popularity of PonyGE2. This will provide a sustainable user base who will contribute to testing and feature development.
